# Comparing Two Methods for the Isolation of Exosomes

**DOI:** 10.1155/2022/8648373

**Published:** 2022-10-25

**Authors:** Mohammad A. Aziz, Benedict Seo, Haizal M. Hussaini, Merilyn Hibma, Alison M. Rich

**Affiliations:** ^1^Sir John Walsh Research Institute, Faculty of Dentistry, University of Otago, PO Box 56, Dunedin 9054, New Zealand; ^2^Department of Pathology, School of Medicine, University of Otago, PO Box 56, Dunedin 9054, New Zealand

## Abstract

Exosomes are membrane-bound nanovesicles released by cells into their extracellular environment. They carry different types of RNA including mRNA which may be useful in the diagnosis of various diseases. Exosome isolation has been a challenge because of their small size; therefore, two exosome isolation methods were compared in this study. The Exoquick-TC PLUS™ exosome isolation kit (kit) was compared with the classic ultracentrifugation (UC) method for exosome isolation. In samples obtained using both methods, cryo-electron microscopy showed round or slightly elongated vesicles with diameters ranging from 50 to 150 nm and delimited by a bilayered membrane. Dynamic light scattering resulted in multiple peaks for kit exosomes, whereas a single peak was observed for UC exosomes. Significantly, more total RNA was present in UC exosomes in contrast to kit exosomes (*P* < 0.0001). This was reflected in subsequent mRNA analysis using qPCR, where UC exosomes had lower Ct values compared to kit exosomes. In conclusion, exosome characterization revealed the presence of exosomes in both UC and the kit samples. The kit samples presented additional peaks from DLS which might be due to impurities. Overall, due to a higher total RNA and mRNA content, UC is a better option for subsequent mRNA analysis; nevertheless, the kit can still be used if an ultracentrifuge is not available as four out of the five genes selected were detected and quantified using the kit.

## 1. Introduction

Exosomes are extracellular vesicles with diameters ranging from 50 to 150 nm [1]. They play important roles under both normal and pathological conditions. The contents of the vesicles may vary in different circumstances [2] and analysis of the contents of exosomes present in body fluids may be used to gather information about the state of the body. Analysis of nonsolid biological tissue, such as exosomal contents, is referred to as a liquid biopsy; it is convenient both for the clinician and for the patient as its collection does not require complicated techniques or any technical skills, and for the patient, it does not result in a wound, nor does the patient have to worry about undergoing surgery as is the case with tissue biopsy. However, it is dependent on the validation and reproducibility of the background science.

The management of patients with oral cancer is a tremendous worldwide liability. Despite significant efforts, the worldwide five-year relative survival rate from oral cancer is generally less than 50% [3]. This low survival rate has remained unchanged for many years despite advances in surgical, radiotherapeutic, and/or medical intervention [4]. Early diagnosis of oral cancer is associated with improved survival; however, the invasive nature, technical requirements, and skill required to perform intraoral biopsies limit their usefulness as a part of community oral cancer screening [5].

The use of saliva or blood-based diagnostic testing to assess the presence of established biomarkers would be advantageous if it could be shown to be a reliable method to detect early-stage tumours and identify high-risk patients [6]. The discovery of exosomes in saliva has provided a pathway to detect oncogenic biomarkers protected within salivary exosomes [7] and the same applies to blood exosomes. It has been established that the major cargo within exosomes is RNA [8]; therefore, exosome yield is considered to be directly proportional to the total RNA obtained. In order to show that analysis of exosome contents is a reliable and effective method for detecting oral cancer biomarkers, a method that yields enough exosomes and in turn yields enough total RNA to detect transcripts of genes within the exosomes using qPCR has to be determined.

Exosomes derived from OSCC have been shown to differentially express mRNAs when compared with healthy controls (Yap et al., 2020). To date, there are no established OSCC biomarkers that can be reliably used to determine the presence of OSCC in body fluids, including blood and saliva in OSCC patients. In an effort to discover OSCC biomarkers in exosomes derived from OSCC cell lines, the genes of interest FOXM1, DNMT1, HOXA7, CCNB1, and HSPA1 were chosen. These have been reported to induce genomic instability and have been proposed to serve as a “first hit” (Teh et al., 2010) in OSCC. These genes are all overexpressed in oral cancers and are linked with early changes in the process of carcinogenesis (Teh et al., 2010). The existence of these genes has not been established in exosomes derived from OSCC. The presence of these in exosomes derived from OSCC is novel and can be used to determine the expression levels in exosomes derived from blood and saliva of patients with OSCC which can be used to detect OSCC at an early stage using liquid biopsy.

The field of exosomes is ever evolving with the greatest challenge being exosome isolation. In order to address important issues like rigor and reproducibility, there is a need to compare new methods with ultracentrifugation which is considered to be the gold standard. The choice of relying on the data of commercial companies which have their own vested interests in these new kits is not recommended. It is essential to come to a conclusion based on an independent comparison done by individuals that do not get any financial or commercial benefits from the product. Therefore, an independent study was conducted to make it easier for new scientists to choose a method for exosome isolation according to their resources and study question. The most classic and accepted method for exosome isolation is ultracentrifugation (UC). This method has several drawbacks, including low reproducibility, low RNA yield, and potential damage to exosomes which can make the technique difficult for clinical utilization [9]. In addition, the method is laborious and time-consuming [10]. The ExoQuick-TC PLUS exosome isolation kit is a relatively new kit that can be used with cell culture medium, saliva, and plasma. The kit does not require any expensive equipment like the ultracentrifuge to operate and is said to be much quicker and less laborious in comparison with UC.

In order to determine a method of choice for exosome isolation, both methods were analyzed using exosomes derived from an *in vitro* representative oral cancer cell line. This included characterization of the exosomes to confirm their nature and assessment of the exosomal cargo, specifically investigating whether or not potential genes of interest (GOI) could be detected.

## 2. Materials and Methods

### 2.1. Cell Culture

The human oral squamous cell carcinoma (OSCC) cell line SCC-4 (ATCC, Manassas, VA, USA) was cultured for 48 hours in Dulbecco's Modified Eagle Medium (DMEM) (Gibco™, Waltham, Massachusetts, USA) supplemented with 400 ng/ml hydrocortisone (Sigma Aldrich, Munich, Germany), 2.5% by volume exosome-depleted FBS one-shot (Gibco™, Waltham, Massachusetts, USA), 1% penicillin/streptomycin (Gibco™, Waltham, Massachusetts, USA), and 0.5% gentamycin (Gibco™, Waltham, Massachusetts, USA). Cell-conditioned medium (CCM) was harvested and centrifuged to remove cells, cell debris, and microvesicles and finally filtered through a 0.2 *μ*m filter and concentrated using Amicon Ultra Centrifugal Filters (Merck KGaA, Darmstadt, Germany) with a 100 kDa cut off. The experiment was repeated three times using three completely independent sets of samples. The concentrated medium was then divided into two and used for exosome isolation using UC and the kit method.

### 2.2. Exosome Isolation

#### 2.2.1. Ultracentrifugation

The protocol for UC was based on previous published research [11, 12] with slight modifications. The concentrated medium was centrifuged at 100,000 × g for 4 h at 4°C (Beckman Coulter, Lane Cove, Australia-Optima XE-90 Ultracentrifuge) in a swinging bucket rotor (Beckman Coulter, Lane Cove, Australia). The supernatant was discarded, and the pellet was resuspended in sterile PBS and centrifuged at 100,000 × g for another 2 hours. The final pellet of exosomes was resuspended in PBS and stored at -80°C until required.

#### 2.2.2. Exosome Isolation Kit

ExoQuick-TC PLUS exosome isolation kit (System Biosciences Inc.; Mountain View, CA, USA) was used following the manufacturer's instructions. The kit consists of prepacked microsphere beads in a column. The CCM is centrifuged, and an exosome precipitation step is carried out. The precipitate is mixed with microsphere beads. The beads are then pulled down through proprietary methodology. The exosomes remain in the supernatant which was then carefully transferred to a new tube and stored at -80°C until required.

#### 2.2.3. Cryo-Electron Microscopy

The exosome preparations were directly deposited onto glow-discharged C-flat 2/2-2C grids (Protochips, Morrisville, NC, USA). Grids were plunge-frozen in liquid ethane using a Vitrobot Cryo Fixation Device (FEI, Hillsboro, OR, USA). Grids of cryo-fixed preparations were imaged on a JEM-2200FS transmission electron microscope (JEOL Ltd., Tokyo, Japan) operated at 200 kV and fitted with a DE-20 direct electron detector (Direct Electron LP., San Diego, USA). Zero-loss image stacks were recorded at −3 *μ*m under focus, with an electron dose of ~5e/Å2 and a nominal magnification of ×25,000 and a pixel size of 0.23 nm. Stacks were aligned (using dose-weighting and patch options) using DE scripts (Direct Electron LP., San Diego, USA) and Motioncorr2 [13].

#### 2.2.4. Dynamic Light Scattering

Fractions of exosomes diluted 1 : 1000 with molecular grade water to a total volume of 1 ml for use in a Zetasizer Nano ZS instrument (ZEN3600, MAL500457, Malvern Instruments, Worcestershire, UK). The experiment was run using three completely independent sets of samples at 4°C, with standard settings, and particle size was calculated as intensity per cent to diameter in nanometer (nm).

### 2.3. Analysis of Exosomal Cargo

The total RNA content between exosomes isolated using both techniques was compared as per methods previously described [14]. qPCR was performed to detect and quantify the selected GOI.

#### 2.3.1. Quantitative PCR

Total RNA was extracted from exosomal pellets using miRNeasy Micro Kit (Qiagen© -217084) following the manufacturer's instructions. The RNA was quantified using the Qubit RNA HS Assay Kit (Qubit™-Q32852) following the manufacturer's instructions with modifications as proposed by Li et al. [15]. Conversion of mRNA samples to cDNA was performed using Transcriptor First-Strand cDNA Synthesis Kit (Roche Diagnostics-04896866001). Preamplification was performed using TaqMan® Preamp Master Mix (TaqMan®-4391128) following manufacturer's instructions. qPCR experiments were performed using TaqMan single-tube gene expression assays for FOXM1(Hs01073586_m1), DNMT1 (Hs00154749_m1), HOXA7 (Hs00600844_m1), CCNB1 (Hs00259126_m1), and HSPA1A (Hs00359163_s1). GAPDH (Hs99999905_ml) was used as a housekeeping gene to normalize the GOI. The qPCR instrument (Roche-LightCycler® 480 Instrument II, Penzberg, Germany) was run with a preincubation of 50°C for 2 min and 95°C for 20 seconds followed by 40 cycles of 95°C for 3 seconds and 60°C for 30 seconds. Data were recorded at 60°C in the cycle. Ct values were recorded, and Microsoft Excel was used for calculation of 2^−∆Ct^ values.

#### 2.3.2. Statistical Analysis

The mean total RNA values were compared between the two groups. Significance was calculated using a two-tailed Student's *t*-test. For comparisons, a value of *P* < 0.05 was considered to indicate a significant difference. All statistical analysis was performed using GraphPad Prism version 8.0, GraphPad Software, La Jolla California USA, http://www.graphpad.com.

## 3. Results

### 3.1. Confirmation by Cryo-EM of the Presence of Vesicles with Sizes Similar to Exosomes

**Figure 1:**
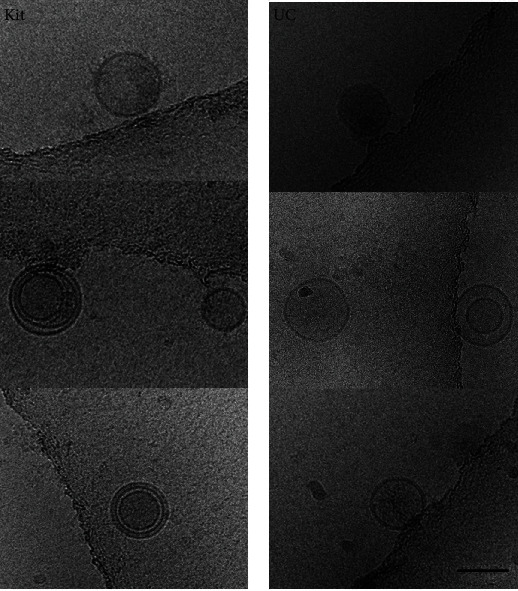
Cryo-EM micrographs. The images show vesicles with sizes similar to exosomes isolated using the kit (a) and UC (b). The vesicles are round and bilayered. The images were obtained at 200 kV on a direct electron detector. Images were recorded at 3 *μ*m under focus, with an electron dose of 15e/Å2 and a nominal magnification of ×25,000 (*n* = 1) (scale bar 100 nm) (OMNI-Electron Microscopy, University of Otago).

Exosomes were isolated from CCM, harvested and centrifuged to remove cells, cell debris, and microvesicles and finally filtered through a 0.2 *μ*m filter and concentrated using Amicon Ultra Centrifugal Filters with a 100 kDa cut off. The experiment was repeated three times using three completely independent sets of samples. The concentrated medium was then divided into two and used for exosome isolation using UC and the kit method. Using cryo-electron microscopy, we were able to visualize bilayered round to slightly elongated shaped vesicles with morphology and size compatible with exosomes (Figure 1).

### 3.2. Results Verified Using Dynamic Light Scattering

**Figure 2:**
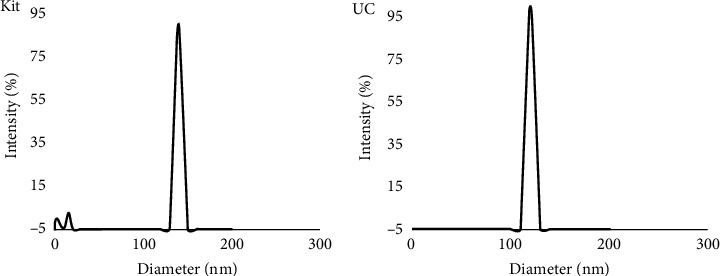
Zetasizer graph showing the size distribution by intensity. One prominent peak (149.9 ± 15.17 nm) and two small peaks (15.2 nm and 5.1 nm) were observed with samples derived using the kit (*n* = 3) (a). A single peak (124.43 ± 9.90 nm) was observed with samples derived using UC (*n* = 3) (b).

The size of the particles in concentrated CCM exosomes isolated using the two methods was measured using a Zetasizer. The modal size of the particles isolated from concentrated CCM by each isolation method are shown in Figure 2. Exosomes isolated using the kit yielded in modal particle sizes in the range 149 ± 15.17 nm, while exosomes isolated by ultracentrifugation had slightly decreased modal particle sizes of 124.43 ± 9.90 nm. The smaller peaks observed in EVs isolated using the kit could be due to protein aggregates.

### 3.3. More RNA Was Present in UC Samples Compared with Kit Samples

Total RNA was extracted from the kit and UC exosomes and measured using a Qubit RNA high-sensitivity assay kit using a spike-in RNA protocol. The amount of total RNA obtained differed significantly between the methods used and ranged from 173.88 ng using UC to 111.25 ng using the kit. The higher yield of RNA in UC exosomes may be due to a higher yield of exosomes in the UC pellet. The kit uses a precipitation method which might not capture all the exosomes in the medium resulting in a lower exosome count indicated by a lower total RNA in the kit samples.

### 3.4. Transcripts of the Genes of Interest Were Detected in Both Samples

The expression of the GOI was determined by qPCR. The results were normalized using the housekeeping gene GAPDH across different samples. Out of the five GOI, FOXM1 was the most highly expressed followed by CCNB1 and HSPA1A. DNMT1 and HOXA7 expression level was very low among all the samples while HOXA7 was not detected in the kit samples. It is interesting that the expression level among all the UC and kit samples is similar. The overall Ct values of the transcripts of the UC samples were less compared to the kit method (Table 1) indicating a higher concentration of individual genes in UC samples.

## 4. Discussion

Despite intense research interest in exosomes in recent years, their isolation remains complex and technically difficult due mainly to their small size and lack of specific surface markers. Various isolation methods and protocols have been reported in the literature, including methods that use UC, precipitation-based methods, and size exclusion chromatography [16].

The ExoQuick-TC PLUS exosome isolation kit, the kit used for this project, is said to be an improved version of the ExoQuick kit and is claimed by the company to be more efficient in isolating exosomes [17]. The original ExoQuick kit has been compared to UC previously, where it was found that exosomes isolated using UC were purer [18]. The kit takes much less time to isolate exosomes and does not require expensive equipment like the ultracentrifuge to operate. The company claims that the microspheres in the improved version of the kit increase the purity of the exosomes, and they also claim that it delivers a higher yield when compared to UC [18]. In the current study, Cryo-EM confirmed the presence of vesicles with sizes similar to exosomes in samples obtained using both methods, as per the definition of exosomes as having a lipid bilayered membrane and being <150 nm in diameter [19]. The Cryo-EM images represent different shapes of exosomes. Some are intact and have a round shape, others have multilayer vesicles, and some demonstrate vesicles within vesicles. All these shapes and forms have been published and are classified as exosomes [20]. The results obtained from the Zetasizer also showed vesicles within the accepted size range for exosomes from both methods. The results from the kit samples had a wide size distribution with a shift towards a larger size compared to UC, and the kit samples showed more than one peak. These additional peaks were considered to be due to impurities, mainly proteins that coprecipitated with exosomes. Others have observed that precipitation-based exosome isolation kits lead to more protein contamination when compared with UC [17], and this is a drawback of this method.

**Figure 3:**
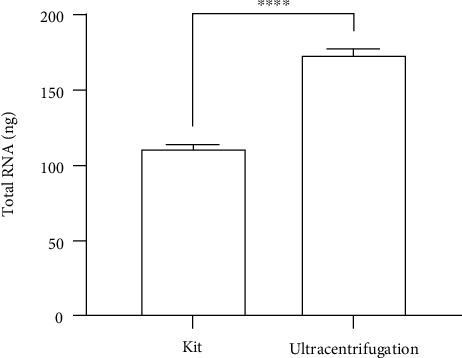
Total RNA obtained using Qubit. The mean total RNA obtained from exosomes isolated by ultracentrifugation was significantly higher (173.88 ± 1.4 ng) (*n* = 3) compared to kit samples (111.25 ± 0.98 ng) (*n* = 3). The *P* value indicates the difference between means of total RNA from exosomes using UC and the kit (^∗∗∗∗^ indicates *P* < 0.0001).

Total RNA quantification is also a challenge when it comes to exosomes. The amount of RNA in exosomes is usually very low, and devices like Nanodrop and Qubit may not be sensitive enough to give reliable results in this situation. Therefore, a protocol was used to increase the quantification limit of Qubit using an RNA spike-in method [15]. Our results showed a significantly higher total RNA yield in the exosomes from the UC group (Figure 3). Higher RNA yields have been reported using the kit, but Nanodrop was used for RNA quantification, which is likely to have overestimated the results [17, 21].

**Figure 4:**
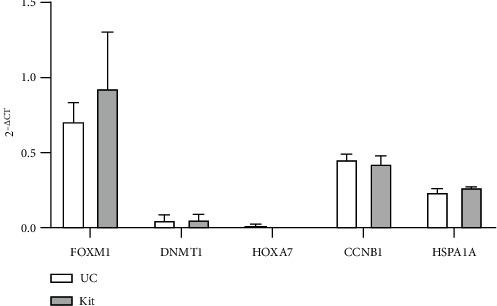
Mean gene expression levels (2^−ΔCT^) from UC and kit samples. All five genes selected for qPCR were detected in samples obtained using UC and the kit except for HOXA7, which was not detected in the kit samples. The data are represented in terms of the mean expression level (2^−ΔCT^) (*n* = 3).

The RNA was reverse transcribed and preamplified, and transcripts of the GOI (FOXM1, DNMT1, HOXA7, CCNB1, and HSPA1A) were quantified using qPCR. The qPCR results showed that the expression levels (2^−ΔCT^) obtained from both UC and kit samples showed a similar trend for all the GOI except for HOXA7, which was not detected in kit samples despite adding a preamplification step (Figure 4). Other researchers have found that different exosome isolation methods show differences in the mRNAs detected [22]. Tang et al. observed that 588 common miRNAs were found in exosome samples isolated using different methods. Exosomes isolated using different methods expressed approximately 200 miRNAs unique to each exosome isolation method [17]. The reason for differences in mRNA and miRNA levels between isolation methods has not been investigated, and it was not clear as to why HOXA7 was not detected in exosomes isolated using the kit. In the UC samples, HOXA7 did however show a relatively lower expression level compared to the rest of the GOI. The lower expression of HOXA7 in the exosomes derived from the cell line might have been the reason that HOXA7 was not detected in the kit samples. The kit samples showed relatively higher Ct values when compared with UC samples indicating that there was a lower copy number of individual genes in kit samples.

In conclusion, the exosome isolation kit has advantages over the UC method as the time and effort required to isolate exosomes are much less; however, UC remains the gold standard since it is more cost-effective and yields a significantly higher total RNA and subsequently a higher mRNA as seen with qPCR. Besides, the exosome samples were purer compared to the kit samples.

## Figures and Tables

**Table 1:** Mean Ct values of kit and UC samples.

FOXM1	DNMT1	HOXA7	CCNB1	HSPA1A	GAPDH
8.81 ± 0.48	11.70 ± 0.18	14.01 ± 0.09	9.26 ± 0.26	10.51 ± 0.44	8.05 ± 0.13
11.40 ± 0.64	14.74 ± 0.24	>40.00	11.98 ± 0.01	12.54 ± 0.19	10.61 ± 0.22

*f*∗mean Ct values ± standard deviation.

## Data Availability

The data is available on request to Dr. Mohammad Aziz, email mohammad.aziz@postgrad.otago.ac.nz, Department of Oral Diagnostic and Surgical Sciences, Faculty of Dentistry, University of Otago, New Zealand.
